# Dr. Henry Woodward Jones

**Published:** 1913-03

**Authors:** 


Dr. Henry Woodward Jones, Detroit Medical College, 1874, died at his home in White Plains, N. Y., January 14. of diabetes, aged 65.

